# Ethical considerations in the globalization of medicine – an interview with James Giordano

**DOI:** 10.1186/1741-7015-11-69

**Published:** 2013-03-14

**Authors:** James Giordano

**Affiliations:** 1 Center for Clinical Bioethics, Georgetown University Medical Center, 4000 Reservoir Road, Washington, DC, 20057, USA

## 

Introduction

**Figure 1:**
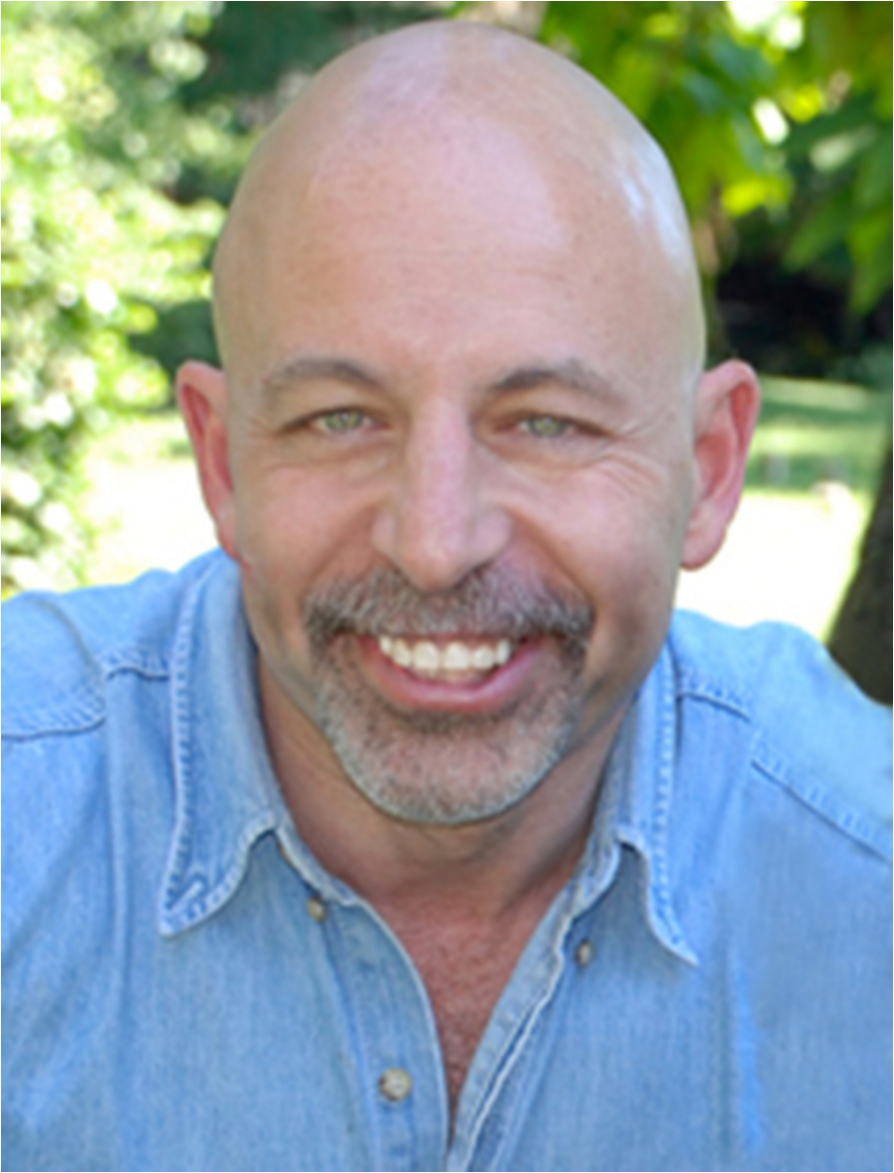
James Giordano.

Prof. James Giordano is Chief of the Neuroethics Studies Program in the Center for Clinical Bioethics, and is on the faculty of the Division of Integrative Physiology, and Graduate Liberal Studies Program at Georgetown University, Washington, DC, USA. He is Clark Fellow in Neurosciences and Ethics at the Human Science Center of Ludwig-Maximilians Universität, Munich, Germany, is 2012–2014 William H. and Ruth Crane Schaefer Distinguished Visiting Professor of Neuroethics at Gallaudet University, Washington, DC, and is also a Senior Fellow of the Potomac Institute for Policy Studies - a Washington-DC area think tank dedicated to the assessment of emerging developments in science and technology, and the ethico-legal and social issues they foster (Figure 1).

As a neuroscientist and neuroethicist, his ongoing research includes the neuroethics of pain research and treatment, and also focuses upon the ways that neuroscience and neurobiotechnology research are conducted in various cultures. Prof. Giordano's work addresses the potential for leveraging scientific and technologic power in - and over - marginalized populations, and the possibility to exert "biopower" through neuroscientific and neurotechnological capability in regional and global economics and social structures. Also, Prof Giordano's studies assess the limitations of neuroscience and neurotechnology, the persistent unknowns inherent to neuroscientific research, and the problems of informed consent and obtaining clinical equipoise when utilizing these approaches in clinical practice. His research group is working to both evaluate the validity and value of existing approaches to neuroethics, and to engage discourse toward developing a more cosmopolitan approach to neuroethics that reflects, and could be viable for use in the evermore diverse world-culture of the 21st century.

In this Q&A, we talk to Prof. Giordano about some of the most important ethical problems that should be considered when conducting biomedical research in all types of clinical settings, with particular emphasis on some of the main challenges of research in low-and-middle-income countries.

The podcast for this interview is available at: http://www.biomedcentral.com/sites/2999/download/Giordano.mp3.

Edited transcript

1. What, in your opinion, are the most important ethical considerations that need to be taken into account when conducting medical research that involves human interventions in particular?

In many ways, this question evokes the spirit of bioethics as first described by philosopher Fritz Jahr some 87 years ago. Jahr's concept, based in part upon the work of his contemporary, the physician/philosopher Albert Schweitzer, and derived, at least to some extent from the ideas of Immanuel Kant, was to treat living beings as ends unto themselves, and not merely as means (to the acquisition of information, and knowledge, etc.). Clearly, this harkens to the tenor of the Nuremberg Code, the Declaration of Helsinki, and the Belmont Report. Taken together with the subsequent work of Tom Beauchamp, and colleague James Childress, these documents concretized the core maxims of beneficence, non-harm, and respect for the autonomy of those who are the subjects of research. While the specific question addresses human research, mention must be made of core principles of beneficence and non-harm in animal research as well - a point becoming ever more relevant in light of an increasing body of neuroscientific research that is demonstrating the cognitive capacity of animals, and the use of neurocentric criteria as the basis of moral regard and treatment of non-human organisms (both in research, and more generally in a variety of other contexts, such as companion animals, livestock, etc.). This is the focus of the work of Sherry Loveless of our group, who is collaborating with colleagues from the Animal Behavior and Conservation Program at the City University of New York to articulate a more finely-grained (neuro)ethics of animal welfare and care. A number of papers in *Philosophy, Ethics and Humanities in Medicine* have addressed the ethics of animal research, as well.

But, to return to the main issue of human experimentation - and particularly clinical trials, the key issues center upon the notion of "good" that is derived from research. We can view science as one of the major pillars of medicine, with research - and perhaps most importantly in the contemporary view, the clinical trial - as the fundamental "tool" with which to acquire scientific knowledge and technical capability. In this way, biomedical science fortifies the knowledge-base and technical capabilities of medicine, and by extension, is instrumental to the achievement of the fundamental goods of health, wellness and a lessening of the human predicament of injury, disease, illness and suffering.

But here it is important to note that the other pillar of medicine is humanitarian consideration and care. I am fond of quoting Edmund Pellegrino's claim that medicine is "…the most humanitarian endeavour of the sciences, and the most scientific endeavour of the humanities".

Indeed, medicine - and the science that serves it - is focused upon the primacy of the good of the person who is the patient. It becomes evident that any discussion of the "good" opens a Pandora's Box when attempting to define what this means in and between peoples, groups and cultures. In clinical research, the (basic) goods and the explicit goals are to generate information necessary to address questions about the nature of a disease, injury and/or illness; if and how a treatment works, in whom, under what conditions, and ultimately, to effect some therapeutic benefit - and to avoid or lessen harms and burdens - to the populations represented by those studied. Yet, questions, issues and problems arise relative to who should be included in research, methods of groups selection, distribution of treatments to those studied, and openness in defining the parameters of the study, its benefits and risks. Of particular emphasis is the notion of informed consent, given the novelty - and in many cases uncertainty - of specific cutting-edge interventions and technologies. This then generates concerns about asymmetries - and inequities - of power that exist between those conducting (and funding) research, and those that are participating subjects, the economic - and in some cases political - forces that may create pulling trends over the scope, conduct and allocation of scientific and medical studies, goods and resources, and how these power dynamics must be addressed, and biomedical research and its translational provisions defined, guided, and governed - both by ethics and through well-informed policy.

2. Does medical research in low-and-middle-income countries present specific ethical challenges?

Very much so. The populations of developing and non-developed countries are often profoundly affected by the plight of disease, injury and impoverishment. These populations have dire need for modern medical care. At face value, this two-fold circumstance of high morbidity and strong need could be viewed as well-defined opportunities to engage research trials of existing interventions (for example, types of comparative effectiveness studies), and new developments in biomedical techniques, tools and technologies. In many cases, this is, in fact, correct. However, it is important to examine the asymmetrical relationships that exist under these conditions. While it could be assumed that conducting research in and with these populations would provide some appreciable benefit to those studied, the question arises whether the beneficial results, outcomes and products of such research will be afforded to these populations, to what extent, and at what cost(s)?

Obviously, a major concern is whether the interventions, goods and services will be sustainable in the host country. Ideally, this would be the case. Yet, if such sustainability involves the provision of goods, resources and/or services by a developed country (rather than a more microeconomically-sensitive sustainability that fosters production and delivery of such goods and resources by the host country itself), then inter-dependency is generated. This can create what French philosopher Michel Foucault referred to as "biopower" - the leveraging of biological factors to evoke dominance and or incur dependence. When incurred on a national scale, this is considered to be a form of "biopolitics", in which the schism(s) between "have" and "have not" countries is economically, socially, and politically manipulated.

The real question then, is how to engage research in these populations, with these populations and for these populations in ways that are sustainable, and at the same time, supportive of the economic infrastructure of the research enterprise. My colleague, economist and sociologist Roland Benedikter of Stanford University, and I have attempted to address these issues, and define potential systems of conducting research that is macroeconomically stable, yet sensitive to and supportive of microeconomic variables and the need for non-dependent sustainability. One of the persistent problems in any such approach is the nature of global markets to move toward supply side dynamics, and the lure of macroeconomic power capability. Increasingly, this is not solely the province of Western nations, but is becoming a more globally relevant variable, given the rise of scientific and technological capabilities of countries such as China, India, and Korea, and the ability of (non-state) individual actors and venture capital groups to develop viable "stakes" in the bioscience and technology markets and at international bargaining tables.

Thus, I believe that we are facing what Professor Benedikter refers to as "A New Global Shift" in the power-dynamics of the 21st century, and biomedicine will play an important role in defining the power balances of this new order. This imparts even greater responsibility to maintain a culturally-sensitive ethical framework for research as the balance of power moves to a more widely distributed domain that is more prominently influenced by non-western cultures, philosophies, needs, values, and ideals. Working in our group, Misti Ault Anderson is specifically addressing what this "Global Shift" may mean for the scope, conduct, and translation of biomedical research upon such a progressively more pluralist world-stage.

3. You mentioned that because of cultural and regional differences, research procedures - such as assuring informed consent - can become more complex. How so?

As previously mentioned, research is being conducted - and highly scrutinised - in a number of developing and non-developed countries. Moreover the contexts of such research are changing. Thus, there is a need to address not only nations and cultures' needs, values and mores, but those ethical ideals and systems that are operative in the countries and cultures in which said research is being undertaken. So, while it is important to ensure that research is conducted ethically, an equally- if not more -important question is "by which ethical standards?" Prior studies by Adriana Petryna support that not attending to variance in cultural values and ethics can deepen inequalities and inequities, and may incur a host of ethical transgressions. Misti Ault Anderson and I have argued that this diversity demands more than superficial appreciation of differing ethical concepts and practises, as there are real risks of adverse consequences if such socio-cultural considerations in the conduct of research are not fully appreciated and recognised. On one hand is the risk of assuming an exclusively western stance, and in so doing committing ethical imperialism. On the other, there is risk of ethical relativism, in treating all situations as *prima facie* contingencies. We believe that both situations risk incurring forms of biopower - in the first instance through cultural dominance, and in the second, through *laissez faire* practices that could overlook certain violations of fundamental goods and human rights simply for economic gain.

Balancing the need for cultural sensitivity and defined ethical guidance demands re-examination of extant ethical systems, principles and codes. Working with philosopher John Shook of the University of Buffalo, and Ernst Pöppel of the Ludwig Maximilians University, Munich, Germany, our group is attempting to develop a system of ethical analysis and revision in order to allow a more cosmopolitan approach that regards anthropological similarities and distinctions, standpoints and dialectic re-address and re-formulation of the moral constructs and standards necessary to inform research and the policies that guide and govern its applications in and across cultures. Needless to say, this remains a work-in-progress.

4. Many clinical researchers are required to work closely with pharmaceutical companies in order to facilitate research. Some argue this introduces a conflict of interest, while others may argue that as long as they are transparent about such associations, this is not a true conflict. What are your views on this?

I would assert that the issue here is not simply limited to associations with pharmaceutical companies. While so-called "big pharma" has been strongly criticised for directionally influencing the conduct and outcomes of research, I think that it's unwise to throw away the baby with the bathwater. Unrestricted corporate grants can- and have - been important and instrumental to conducting and supporting research that has led to a number of significant and valuable findings and products. Yes, ethical improbity has occurred in a number of research scenarios, but the pharma-academic association is not unique in this regard. Be that as it may, there is a crucial lesson to be learned from the scrutiny of, and re-posturing toward pharmaceutical support of academic research. Namely, it has prompted increased diligence in assessing and reporting conflicts of interest, and a more reputable research infrastructure that conjoins the academic, commercial and governmental sectors (what has become known as the "triple helix" of the contemporary scientific and technological research enterprise).

This is important given the very real circumstances of shrinking federal research funding (in light of continuing resolutions and a potential sequestration crisis). Private foundations and the commercial sector can and likely will fill the gap generated by the decrease, if not loss, of federal subsidies. I do not see this as negative; to the contrary, I believe, or perhaps more accurately hope, that this will compel a revision in the way(s) that research is approached and executed. To be sure, there has been defensible criticism against research endeavours that were overly agnostic, or aimed solely at large scale market shares. A revisionist stance could re-direct research toward obtaining more "medicine-based evidence", and developing outcomes, techniques and products that translate into care for smaller, more focused market sectors. This would marry well with incentives for personalised medicine. Yet, there is an equally defensible argument against corporate funding of academic research in that it is seen as "purloining" the integrity of the work, and inducing coercive influence in the scope and outcomes of the results. In principle this is a valid concern. However, scrutiny and insights gained over the past decade have levied against this situation. It may be that from these ashes may rise a phoenix that positions the commercial sector to more diligently support academic research, and in so doing, allow for sustained engagement and innovation through these times of economic hardship.

5. How can editors as well as clinical researchers ensure that all medical research is conducted ethically?

I see editors as collaborative partners, working with IRBs and researchers, to ensure quality reporting and dissemination of findings, ideas and critique. I like to tell my students that scholarship is one of the only fields in which one is "guilty until proven innocent". The peer-review and editorial process establishes this gauntlet of demonstration of "innocence" that the work, as reported passes the muster of skepsis and scrutiny. The requirement for authors to affirm that any and all research was conducted with IRB approval, and in accordance with internationally accepted guidelines and standards represents a major step in re-grounding researchers' responsibility for the ethical conduct of their work. While good reviewers often cite apparent problems in the ethical aspects of a particular study, editorial diligence and responsibility cannot be lax. In many ways, the editor serves as the first - and perhaps final - threshold that must be crossed when evaluating the potential ethical conduct of research as reported in a given manuscript. Of course, there will always be variation in the quality of papers, and results. Yet, I think that the open access format is an asset in that it creates a more extensive forum for the dissemination of findings, and in this way, enables a larger audience of both readership and criticism.

6. You are a neuroscientist and neuroethicist. What do you see as the major ethical issues in neuroscientific research at present?

Neuroscientific and neurotechnological research incur all of the same ethical issues as any other discipline of cutting edge biomedical science: protection of subjects, consent, conflicts of interest, are all germane to the responsible conduct of neuroscientific studies. However, I think that neuroscience evokes a number of unique issues and questions that reflect the nature and focus of the field itself. First, I see these as stemming from the as yet unanswered, so-called hard question or problem of the field, namely how consciousness or mind occurs in brain. Second, I see ethical issues spawned by the intersection of unknowns: how the brain actually functions (on the level of efficient causality), the nature of normality and abnormality, the novelty of various techniques and technologies, and how these techniques and technologies affect the structure and function of the brain to exert effects (in treatments, enhancements, etc.). Third, are those ethical issues generated by the use and/or misuse of neuroscience and neurotechnologies in a range of social applications, including medicine, the public sphere - to change norms, standards, values and human relationships, and in national security and defense.

The fundamental ethical questions are what should we do with all the neuroscientific knowledge and capabilities we possess, and what should we do about the knowledge and capabilities we lack? This speaks directly to what is sometimes referred to as the "first-tradition" of neuroethics: the study of the possible neurological bases of morality, ethics and relational beliefs and actions. In this way, neuroethics may be seen as a type of meta-ethics that can help to understand how and why we have certain moral cognitions, beliefs and develop particular ethical systems for guiding our actions. But, then we must ask - can neuroscience and its technologies actually afford this knowledge, and if so, how should we use this knowledge to affect the human condition? Obviously, I'll be at this for a while…these are not easy questions, and there are no simple answers.

7. Where can I find out more?

Basic Research Ethics:

Brody B. *The Ethics of Biomedical Research*. NY: Oxford University Press, 1998.

Council for International Organizations of Medical Science (CIOMS). *International Ethical Guidelines for Biomedical Research Involving Human Subjects*. Geneva: CIOMS and WHO, 2002.

Giordano S. The 2008 Declaration of Helsinki: Some reflections. *J Med Ethics*, 2010, 36(10); 598–603.

Goodman KW. *Ethics and Evidence-based Medicine*. Cambridge: Cambridge University Press, 2004.

Levine RJ. *Ethics and Regulation of Clinical Research*. New Haven: Yale University Press, 1986.

Inter-cultural/Global Ethics:

Anderson MA. Ethical considerations in international biomedical research. *Synesis: A*

*Journal of Science, Technology, Ethics, and Policy*, 2011, 2: G56-61.

Benatar S. Towards progress in resolving dilemmas in international research ethics. *J Law, Med Ethics,* 2007, 32(4): 574–582.

Bowman J. Bioethics and cultural pluralism. *Humane Health Care International* 1997, 13(2); 31–34.

Foucault M. *Security, Territory, Population*. NY: Macmillan, 2007.

Lavery JV. Putting international research ethics guidelines to work for the benefit of developing countries. *Yale J of Health Policy, Law and Ethics*, 2004, 4(2): 319–336.

Ethics of Commercial-Academic and Triple-Helix Enterprises:

Decker M. The role of ethics in interdisciplinary technology assessment. *Poiesis Praxis*, 2004, 27(2): 139–156.

Etzkowitz H, Leydesdorff L. The dynamics of innovation: From national systems and Mode-2 to a triple helix of university-industry-government relations. *Research Policy*, 2000, 29: 109–123.

Wurzman R. Inter-disciplinarity and constructs for STEM education: At the edge of the rabbit hole. *Synesis: A Journal of Science, Technology, Ethics, and Policy*, 2010, 1(1): G32-35.

Ethics and Scientific Publication:

Brackbill Y, Hellegers AE. Ethics and editors. *Hastings Center Report*, 1980, 10(2): 20–22.

*Committee on Publication Ethics* (COPE). Guidelines and cases available at: http://publicationethics.org

Jones AH, McLellan F. (eds. ) *Ethical Issues in Biomedical Publication*. Baltimore: Johns Hopkins University Press, 2000.

Neuroscience, Neurotechnology and Neuroethics:

Giordano J. (ed.) *Neurotechnology: Premises, Potential and Problems*. New York: CRC Press, 2012.

Giordano J, Benedikter R. An early - and necessary - flight of the Owl of Minerva: Neuroscience, neurotechnology, human socio-cultural boundaries, and the importance of neuroethics. *J. Evolution and Technol*. 2012, 22(1): 14–25.

Giordano J, Gordijn B. (eds.) *Scientific and Philosophical Perspectives in Neuroethics*. Cambridge: Cambridge University Press, 2010.

Giordano J, Olds J. On the interfluence of neuroscience, neuroethics and legal and social issues: The need for (N)ELSI. *AJOB-Neuroscience* 2010, 2(2): 13–15.

Loveless SE, Giordano J. Neuroethics, painience and neurocentric criteria for the moral treatment of animals. *Cambridge Q Healthcare Ethics* (in press; preprint available upon request at http://www.neurobioethics.org).

